# Lacrimal Intubation in the Management of Congenital Nasolacrimal Duct Obstruction: A Prospective Comparative Study

**DOI:** 10.1155/joph/4709302

**Published:** 2026-05-18

**Authors:** Haitham Thabit Rashdan, Mohamed Salah Hamed Korishy, Hany Mahmoud, Alaa Abdalsadek Ahmed Sinjab, Islam Awny

**Affiliations:** ^1^ Ophthalmology Department, Sohag University, Sohag, Egypt, sohag-univ.edu.eg

**Keywords:** congenital nasolacrimal duct obstruction, endoscopic, epiphora, lacrimal intubation

## Abstract

**Background:**

Congenital nasolacrimal duct obstruction (CNLDO) is a common cause of epiphora in children. Lacrimal intubation is often performed when probing fails, but the optimal approach remains debated. This study compared the outcomes of nasal endoscopy–guided and blind closed lacrimal intubation in pediatric patients with CNLDO.

**Methods:**

This prospective comparative study (single‐center retrospectively registered) was conducted on 63 eyes of 39 children under 16 years. Participants were randomly assigned to blind closed intubation (*n* = 31) or nasal endoscopy–guided intubation (*n* = 32). Outcomes included operative time, epiphora score, fluorescein dye disappearance test (FDDT), complications, and patient satisfaction.

**Results:**

Operation time was significantly lower in the nasal endoscopy–guided group than that in the closed group (*p* < 0.001). Epiphora score, FDDT, postoperative complications, and satisfaction were comparable. Cut tube occurred in 3.23% of the blind group versus 3.45% of the nasal endoscopy–guided group, while cheese‐wiring and persistent tube were observed in 3.23% and 6.9% of the blind cases compared with 6.9% and 6.9% of the nasal endoscopy–guided cases, respectively. Prominent tube and postoperative lacrimation were noted only in the nasal endoscopy–guided group (13.79% and 6.9%), whereas dacryocystitis and recurrence were confined to the blind group (6.45% and 12.9%). Regarding satisfaction, high satisfaction was reported in 70.97% of the blind group versus 59.38% of the nasal endoscopy–guided group, moderate satisfaction in 12.9% versus 31.25%, and no satisfaction in 16.13% versus 9.38%, respectively.

**Conclusions:**

Both blind and endoscopic lacrimal intubation achieved high success rates in children with CNLDO. The nasal endoscopy‐guided technique shortened operative time and offered better visualization, but overall outcomes and satisfaction were comparable.

**Trial Registration:** Pan African Clinical Trials Registry (PACTR): PACTR202510699342440

## 1. Introduction

Approximately 20% of all neonates are affected by congenital nasolacrimal duct obstruction (CNLDO). The CNLDO condition is defined by the obstruction of the distal end of the nasolacrimal duct (NLD). This obstruction is typically the result of the persistence of a mucosal membrane known as Hasner’s membrane or developmental abnormalities in the adjacent bone structures, which obstruct the nasal lacrimal drainage system [1].

The initial treatment for CNLDO is based on conservative management. The high rate of spontaneous resolution in neonates is primarily responsible for the effectiveness of techniques such as lacrimal sac stimulation and probe in approximately 90% of the cases treated within the first year of life [2]. Nevertheless, surgical interventions are frequently necessary in complex cases that involve congenital malformations or recurrent infections [3].

Lacrimal intubation is a surgical intervention that is frequently employed to treat CNLDO in infants, particularly when conservative treatments are unsuccessful [4]. A silicone catheter is inserted into the lacrimal outflow system during lacrimal intubation to prevent secondary stenosis and restore patency [5].

By mechanically dilating the lacrimal passages and allowing tears to travel over its surface through capillary action, stent insertion reduces tear flow resistance. The tubes have the potential to rectify various curves in the canaliculi, thereby facilitating the discharge through these confined conduits [6].

Following the insertion of the tube and probing, the introducer can be recovered either endoscopically or blindly via metallic guide or guide wire [7]. Direct visualization during the procedure is facilitated by nasal endoscopic–guided probe, which improves success rates and facilitates the identification of obstruction causes. Despite the tactile feedback that blind maneuvers may offer, they can be more traumatic than the optimized approach of nasal endoscopic guidance [8].

Dacryoendoscopy, which is an emerging minimally invasive technique, showed promising results with the instrumental advances and endoscopic experiences [9]. Nasal endoscopic–guided intubation is more popular.

Comparative investigations on blind versus nasal endoscopy–guided procedures are scarce, resulting in evidence vacuum for clinicians. Consequently, this investigation evaluated the outcomes of lacrimal intubation in the treatment of CNLDO.

## 2. Patients and Methods

This prospective comparative study (single‐center retrospectively registered) was conducted on 63 eyes of 39 patients, aged less than 16 years, of both sexes, who presented with epiphora and were diagnosed with CNLDO. The patients were divided into two groups: the blind closed intubation group (*n* = 31) and the nasal endoscopy–guided intubation group (*n* = 32).

From August 2024 to April 2025, this investigation was conducted with the informed written consent of all caregivers, following approval from the ethical committee of Sohag University Hospitals (approval code: Soh‐med‐25‐3).

Method of randomization: patients were randomly assigned to either blind closed intubation group or nasal endoscopy–guided intubation group using a sealed opaque envelope method. Sequentially numbered envelopes containing the treatment allocation were prepared prior to the start of the study and opened immediately before surgery.

Individuals were precluded if they had a history of trauma, previous surgery in the lacrimal passages, or patients older than 16 years.

All participants underwent a comprehensive medical history, clinical examination, extensive ophthalmological evaluation to rule out comorbidities, meticulous lid and lacrimal examination, and laboratory testing prior to the surgical procedure.

General anesthesia was administered during each surgical procedure. Probing was conducted to verify the diagnosis and rule out canalicular obstruction.

### 2.1. Blind Bicanalicular Intubation Technique

Initially, the lacrimal probing and syringing test was implemented to rule out the presence of concomitant punctual or canalicular stenosis. Then, a Nettleship punctal dilator was used to dilate the punctum. The diameter of the instrument that passed through the punctum ranged from 0.70 to 1.10 mm. As the instrument advanced along the canaliculus, gentle lateral traction was applied to the lid until it reached the nasal bone. The instrument was then rotated 90° and delicately introduced into the NLD before being advanced into the nose.

During probing, a gritty sensation was experienced along the stenotic duct, and in the presence of a distal membrane, a distinct “pop” was perceived upon breaching the membrane. Upon suggestion, and when necessary, the inferior turbinate was medially infractured to expose the underlying region in both groups. Patency of the NLD was verified by many techniques. The probe’s terminus is sometimes immediately seen and palpated with an additional probe, while a tiny bolus of saline, often colored with fluorescein, is irrigated via the duct and then aspirated using suction.

Bicanalicular stents consist of two probes separated by an intervening stent. One probe is inserted via the superior punctum and the other through the inferior punctum. The probes were extracted, and the free ends of the stent were affixed in the nasal cavity, sometimes reinforced with a suture.

### 2.2. Nasal Endoscopy–Guided Intubation Technique

The syringing and probing test was conducted to rule out concurrent punctal or canalicular stenosis. The superior punctum was expanded using a Nettleship punctal dilator. The Bowman’s probe was used for probing purposes. The probe was thereafter inserted vertically into the punctum and twisted horizontally by 90° while laterally retracting the outer canthus in the same plane to access the canaliculus.

The probe was advanced until it contacted the nasal wall of the lacrimal sac and then rotated vertically, being maneuvered gently to prevent the creation of a false channel via the NLD. The nasal endoscopy was conducted in the nasal cavity under the inferior turbinate to evaluate probing via visualization of the inferior meatus. Figure 1.

FIGURE 1Direct visualization of the probe using nasal endoscopy.

**Figure:**
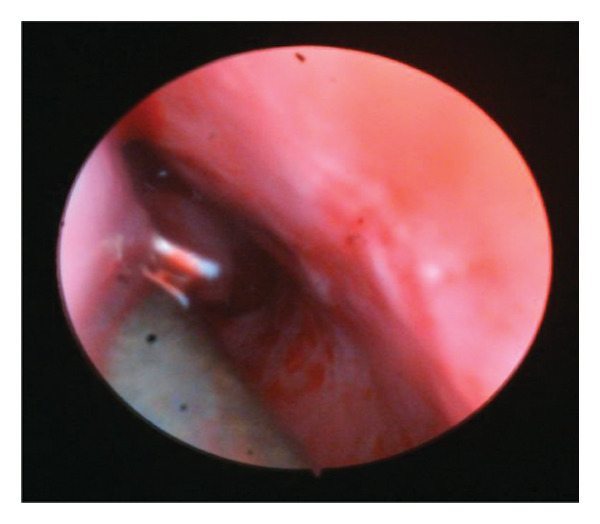
(a)

**Figure:**
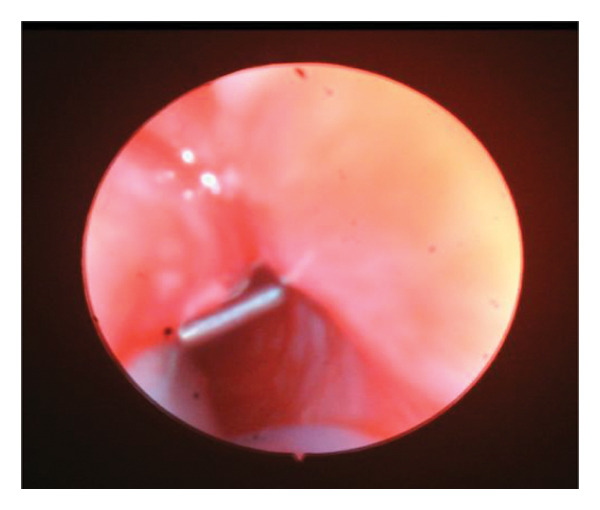
(b)

Topical antibiotic eye drops were administered four times daily, and xylometazoline hydrochloride 0.05% nasal sprays were used for 1 week. The resolution of blockage was characterized by the lack of watering or discharge, accompanied by a normal tear meniscus height and a negative fluorescein dye disappearance test (FDDT).

Postoperatively, participants received a prescription for topical antibiotic eye drops to be administered every 4 hours throughout the first postoperative phase. Subsequently, medication intervals were modified based on surgical discharge and epiphora.

Epiphora was assessed using a combined Munk and epiphora scoring system described by Malet et al. [10]. During follow‐up visits, tearing was graded as follows: 0 for no tearing, 1 for tearing occasionally in windy conditions, 2 for constant tearing sometimes requiring wiping, and 3 for constant tearing always requiring wiping. A slit‐lamp examination was performed to evaluate tear meniscus height, conjunctival vessel congestion, and lower lid skin maceration. Additionally, a FDDT was conducted by instilling one drop of 2% fluorescein and assessing the remaining dye in the tear meniscus after 3 and 5 min, graded as Grade 1 (< 3 min), Grade 2 (3–5 min), and Grade 3 (> 5 min).

Patients were monitored on the first day, after 1 week, 1 month, and 3 months.

The postoperative assessment included the FDDT, complications, enhancement of epiphora, and patient satisfaction.

Surgical success was defined as the simultaneous presence of the following:

• Absence of epiphora or discharge
• Normal tear meniscus height on slit‐lamp examination
• Negative FDDT.

Outcome assessment was performed by the treating team and was not blinded to the surgical technique.

Silicone tubes were planned to remain in place for approximately 3 months and were removed during the follow‐up. Earlier tube removal was done in cases with complications as cut or prominent tube.

### 2.3. Statistical Analysis

Statistical analysis was conducted using SPSS Version 29 (IBM, Armonk, NY, USA). The Shapiro–Wilk test and histograms were used to assess the normality of the data distribution. Quantitative parametric data were expressed as mean and standard deviation (SD) and evaluated using an unpaired Student’s *t*‐test. Qualitative variables were expressed as frequency and percentage and examined with the chi‐square test or Fisher’s exact test if applicable. A two‐tailed *p* value ≤ 0.05 was deemed statistically significant.

## 3. Results

**FIGURE 2:**
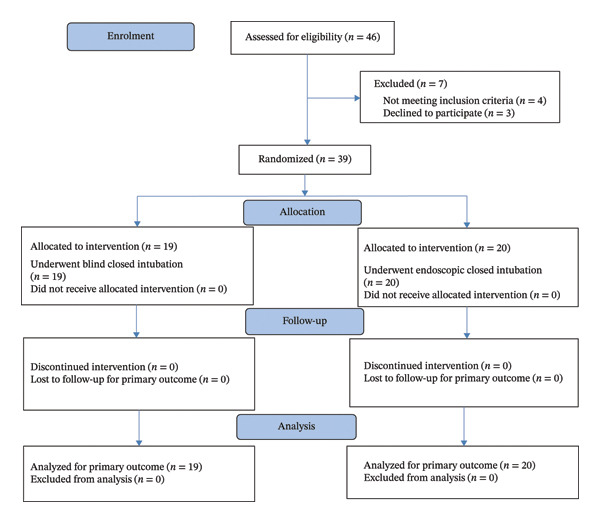
CONSORT flowchart of the enrolled patients.

CONSORT flowchart of the enrolled patients is illustrated (Figure 2).

Age, sex, and intraoperative complications were insignificantly different between both groups. Operation time was significantly lower in the nasal endoscopy–guided group than that in the closed group (*p* < 0.001) (Table 1).

**TABLE 1:** Demographic data and intraoperative complications pivot shift test of the studied groups.

		Blind closed intubation group (*n* = 19)	Nasal endoscopic closed intubation group (*n* = 20)	*p*	Mean difference/RR (95% CI)
Age (years)		5.53 ± 2.63	7.35 ± 3.77	0.090	−1.8 (−3.3:−0.4)
Sex	Male	13 (68.42%)	10 (50%)	0.242	1.4 (0.8:2.33)
	Female	6 (31.58%)	10 (50%)		

		**(*n* = 31)**	**(*n* = 32)**		

Operation time (min)		18.77 ± 4.74	12.66 ± 5.12	< 0.001	−1.8 (−3.3:−0.4)
Intraoperative complications	Difficult intubation	19 (61.29%)	19 (59.38%)	1.00	1 (0.72:1.39)
	Hypertrophic turbinate	2 (6.25%)	2 (6.06%)		
	Bleeding	7 (22.58%)	7 (21.88%)		

*Note:* Data are presented as mean ± SD or frequency (%).

Abbreviation: RR, relative risk.

The epiphora score and dye disappearance test were insignificantly different between both groups (Table 2).

**TABLE 2:** Epiphora score and dye disappearance test of the studied groups.

		Blind closed intubation group (*n* = 31)	Nasal endoscopic closed intubation group (*n* = 32)	*p*	RR (95% CI)
Epiphora score	Always tearing and need to wipe	4 (12.9%)	4 (12.5%)	0.803	1.2 (0.33:4.01)
	Tearing sometime in windy days	12 (37.5%)	10 (30.3%)		
	No tearing	15 (48.39%)	18 (56.25%)		

Dye disappearance test	< 3	16 (51.61%)	14 (43.75%)	0.532	1.18 (0.7:1.98)
	3–5	15 (48.39%)	18 (56.25%)		

*Note:* Data are presented as frequency (%).

Abbreviation: RR, relative risk.

Postoperative complications and satisfaction showed no significant differences between the two groups. Tube cutting occurred in 3.23% of the blind group compared with 3.45% of the nasal endoscopy–guided group although cheese‐wiring and persistent tubes were seen in 3.23% and 6.9% of the blind cases, respectively, vs. 6.9% and 6.9% of the endoscopic cases. Significant tube placement and postoperative lacrimation were seen only in the nasal endoscopy–guided cohort (13.79% and 6.9%), whereas dacryocystitis and recurrence were exclusive to the blind cohort (6.45% and 12.9%). In terms of satisfaction, 70.97% of the blind group claimed great pleasure compared with 59.38% of the nasal endoscopy–guided group, while 12.9% of the blind group indicated moderate happiness vs. 31.25% of the nasal endoscopy–guided group, and 16.13% of the blind group reported no satisfaction compared with 9.38% of the nasal endoscopy–guided group (Table 3 and Figure 3).

**TABLE 3:** Postoperative complications and satisfaction of the studied groups.

		Blind closed intubation group (*n* = 31)	Nasal endoscopic closed intubation group (*n* = 32)	*p*	RR (95% CI)
Postoperative complications	Cut tube	1 (3.23%)	1 (3.45%)	0.072	0.83 (0.06:12.3)
	Cheese‐wiring	1 (3.23%)	2 (6.9%)		
	Dacryocystitis	2 (6.45%)	0 (0%)		
	Prominent tube	0 (0%)	4 (13.79%)		
	Persistent tube	1 (3.23%)	2 (6.9%)		
	Lacrimation	0 (0%)	2 (6.9%)		
	Recurrence	4 (12.9%)	0 (0%)		

Satisfaction	High	22 (70.97%)	19 (59.38%)	0.194	1.36 (0.36:5.0)
	Moderate	4 (12.9%)	10 (31.25%)		
	Not satisfaction	5 (16.13%)	3 (9.38%)		

*Note:* Data are presented as frequency (%).

Abbreviation: RR, relative risk.

FIGURE 3Tube in place 1 week after (a) right‐eye blind closed intubation showing improvement in epiphora, (b) nasal endoscopic closed intubation with symptomatic improvement, (c) bilateral tubes with persistent epiphora 1 month postoperatively, and (d) torn tube 1 week after blind closed intubation with recurrent tearing.

**Figure:**
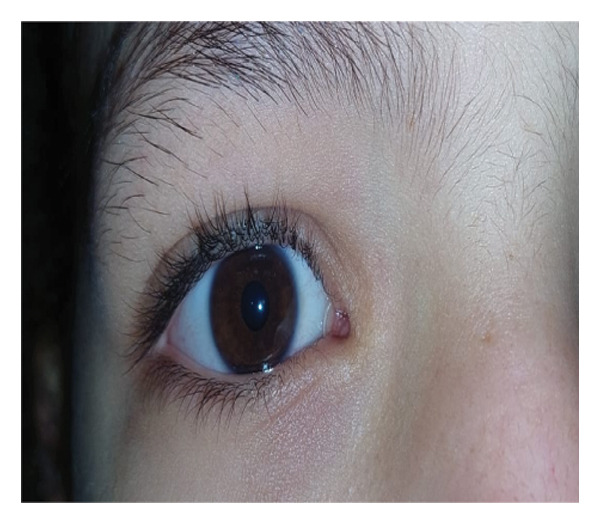
(a)

**Figure:**
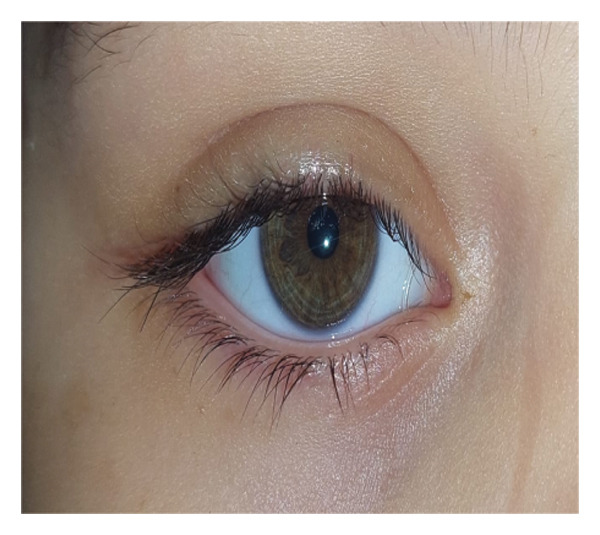
(b)

**Figure:**
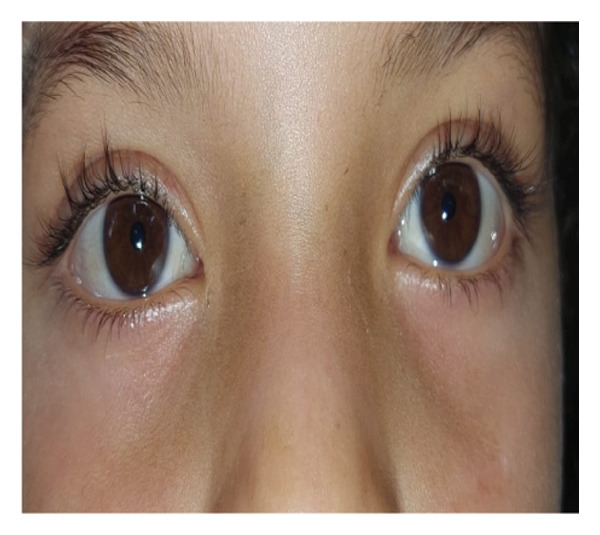
(c)

**Figure:**
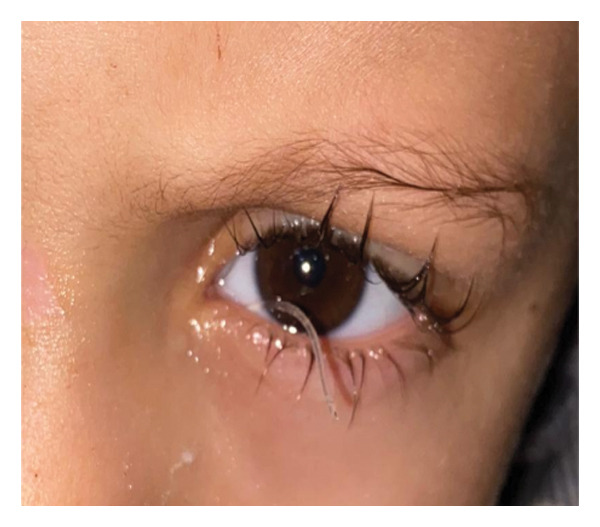
(d)

## 4. Discussion

We observed that nasal endoscopy–guided intubation was much more rapid than blind intubation (18.77 ± 4.74 vs. 12.66 ± 5.12). Both groups obtained comparable alleviation of epiphora and achieved full tear clearing. No significant differences were seen in postoperative complications and satisfaction between the groups. Cut tube incidence was 3.23% compared with 3.45%, cheese‐wiring occurred at 3.23% vs. 6.9%, and permanent tube rates were 3.23% against 6.9% for the blind and nasal endoscopy–guided groups, respectively. Significant tube and lacrimation were seen only in the nasal endoscopy–guided group (13.79% and 6.9%), while dacryocystitis and recurrence were noted solely in the blind group (6.45% and 12.9%). In the blind group, satisfaction levels were rated as high, moderate, and none at 70.97%, 12.9%, and 16.13%, respectively, compared with the nasal endoscopy–guided group, which reported 59.38%, 31.25%, and 9.38% for the same categories.

The reduced operational duration associated with endoscopy certainly indicates the benefit of direct vision. During nasal endoscopy, the surgeon may navigate the probe and stent via the duct, circumventing protracted blind movements. The real‐time visualization of the lacrimal channel, including Hasner’s valve, enhances canalicular navigation and reduces the need for repetitive probing [11].

Conversely, blind intubation relies on tactile feedback, potentially extending the duration of the surgery in cases of anatomical ambiguity. Nasal endoscopy facilitates the instant recognition of anatomical changes, such as a constricted inferior turbinate or osseous blockage, allowing for swift rectification and expedited conclusion. The complication rates were comparably low; however, dacryocystitis and recurrence were exclusively observed in the blind group, indicating that residual membrane or false passage formation during blind probing may elevate the risk of infection or inadequate drainage, whereas nasal endoscopic visualization could mitigate these complications [8, 11].

Similarly, Sharaf et al. [12] showed no significant difference in final epiphora scores or dye‐test outcomes. Sharaf’s team saw a large reduction in postoperative hemorrhage with endoscopy (4% compared with 24% with blind intubation, *p* < 0.05) and a tendency toward fewer recurrences (8% against 12%, not statistically significant).

Consistent with our results, Alruwaili and colleagues [13] indicated that silicone intubation often resulted in a greater likelihood of symptom alleviation compared with probing alone.

Likewise, Singh et al. [14] demonstrated that bicanalicular intubation is advantageous owing to its greater combined diameter, stable knot placement in the inferior meatus assuring sufficient NLD stenting, and enhanced dynamic movement with eyelid blinking.

Al‐Faky et al. [15] concluded that intubation could achieve more successful outcome in complex CNLDO compared with probing. Cases with bony obstruction or craniofacial syndrome need intubation to maintain patent passages and overcome the obstruction.

Similarly, Schellini and colleagues [16] assert that direct nasal sight significantly enhances success by preventing probe misplacement. They contend that nasal endoscopic probing/intubation accurately detects the specific blockage (membranous versus stenotic ostium) and facilitates prompt treatment, hence enhancing the likelihood of therapeutic success and averting iatrogenic false passages. They assert that no indirect test equals the efficacy of endoscopic verification.

Nakamura et al. [5] observed an increased patency rate with larger‐diameter tubes (85.7% for 1.5 mm compared with 73.9% for 1.0 mm), hence reinforcing our selection of tube size (0.7–1.1 mm probes) for sufficient duct dilation.

Also, Hamed et al. [17] observed that blood loss was reduced in the nasal endoscopic group compared with the blind group. Surgical success was attained in 100% of the endoscopic group, while it was obtained with 82% of the blind group.

Similarly, Abd El Ghafar [8] observed a 94.12% success rate for nasal endoscopic–guided probing in infants, concluding that this technique transforms probing from a blind procedure to a visualized one, identifies the cause of obstruction and false passage, and facilitates intraoperative adjustments of false passages, thereby enhancing the success rate.

Consistent with our findings, Espinoza and Lachmund [18] indicated that nasal endoscopy–assisted probing or intubation aids in verifying probe placement and identifying intranasal anomalies that might diminish the success rate.

Rajabi et al. [19] evaluated several stent types in children under 4 years, revealing a superior success rate for bicanalicular tubes (96.4%) compared with monocanalicular varieties (71.5% and 47.3%).

Soltani Shahgoli et al. [20] found that inferior turbinate fracture (ITF), whether combined with probing or performed with intubation, had a minimal impact on outcomes for CNLDO treatment especially in younger children (12–24 months). However, the older children (24–36 months) appeared to derive greater benefit from the procedure. Shahgoli considered the ITF as a separate maneuver capable of achieving favorable results in CNLDO management; notably, when combined with other interventions, it did not significantly alter the success rates. In our study, the inferior turbinate was fractured in selected cases, and this could be a source of bias, we recorded it in limitations.

### 4.1. Limitations

We consider the following limitations in our study. First, the sample size was relatively small; further studies of larger sample size to be considered. Second, the follow‐up period of 3 months evaluated only short‐term outcomes and may not reflect long‐term recurrence rates. Third, outcome assessment was not blinded. Finally, some patients contributed both eyes to the analysis, which may violate the assumption of independence between observations. An important notable limitation is the lack of consistency in surgical techniques, as ITF was applied in one cohort; furthermore, the study was registered retrospectively.

## 5. Conclusions

Both blind and nasal endoscopic lacrimal intubation achieved high success rates in children with CNLDO. Nasal endoscopic guidance shortened operative time and offered better visualization, but overall outcomes and satisfaction were comparable. Blind intubation remains an effective option, while nasal endoscopic guidance adds value in complex or uncertain cases.

## Funding

We have no financial interests.

## Disclosure

The authors declare that there are no additional disclosures to report.

## Conflicts of Interest

The authors declare no conflicts of interest.

## Data Availability

The data that support the findings of this study are available from the corresponding author upon reasonable request.
